# Transversus abdominis plane block for bilateral orchiopexy in an 8-year-old patient with Eisenmenger’s syndrome

**DOI:** 10.1186/s40981-015-0013-6

**Published:** 2015-09-02

**Authors:** Yu Matsumoto, Satoshi Shibuta, Tomotaka Morita, Takeshi Iritakenishi, Nobuyuki Nishimura, Moe Koide, Yuji Fujino

**Affiliations:** Department of Anesthesiology and Intensive Care Medicine, Graduate School of Medicine D7, Osaka University, 2-2, Yamadaoka, Suita, 565-0871 Japan

**Keywords:** TAP block, Eisenmenger’s syndrome, Pediatric anesthesia

## Abstract

Non-cardiac surgery should only be performed in patients with Eisenmenger’s syndrome if absolutely mandatory because these patients are at high risk of perioperative mortality. Proper anesthetic and perioperative pain management in these patients remains a controversial topic. Transversus abdominis plane (TAP) block provides safe and beneficial perioperative analgesia in adults and children; however, no report has described the performance of TAP block in a child with Eisenmenger’s syndrome.

Herein, we describe the performance of bilateral orchiopexy for cryptorchidism in an 8-year-old boy with Eisenmenger’s syndrome due to an uncorrected muscular ventricular septal defect (mVSD). Anesthesia induction and maintenance were uneventful. Subsequently, the patient received ultrasound-guided bilateral TAP block by using 10 mL of 0.25 % levobupivacaine shortly before recovery from anesthesia. The TAP block provided pain relief and maintenance of stable hemodynamics during the postoperative period.

We successfully used a TAP block in a child with Eisenmenger’s syndrome to provide postoperative analgesia. No side effects were apparent during the perioperative period. TAP block can be considered a beneficial pain management technique for analgesia in children with Eisenmenger’s syndrome.

## Background

Anesthetic and perioperative pain management in patients with Eisenmenger’s syndrome is one of the greatest concerns among anesthesiologists because these patients have a serious risk of hypoxia due to right to left (RL) intracardiac shunt causing irreversible pulmonary hypertension. Factors that increase RL shunt are known to worsen arterial hypoxia as well as threaten the patient’s life [1–4]. Postoperative pain is one of the most important factors associated with disturbance of the balance between systemic and pulmonary circulation [3, 5]; therefore, reducing postoperative pain is critically important for patients with Eisenmenger’s syndrome. Herein, we report a noteworthy case in which we provided successful postoperative analgesia by using transversus abdominis plane (TAP) block.

## Case presentation

Bilateral orchiopexy for cryptorchidism was scheduled in an 8-year-old boy (height, 107 cm; weight, 17.2 kg) with Eisenmenger’s syndrome due to an uncorrected muscular ventricular septal defect (mVSD).

He was born at 32 weeks of gestation by caesarean delivery at a weight of 1184 g. After his birth, mVSD and congenital esophageal atresia (type C) were diagnosed. Esophageal closure was performed 4 days after birth. After this operation, the patient required long-term treatment for complications such as chylothorax and infections. In addition, tracheostomy was performed at 1 year of age because of tracheomalacia, and the patient has remained on a ventilator since then. Meanwhile, he missed an opportunity for the surgical justification of mVSD closure with over-systemic pulmonary hypertension evaluated by cardiac catheterization at 17 months of age.

The most recent detailed echocardiography, which was performed when the patient was 3 years old, showed a left to right (LR) shunt during the systolic period (rate, 1.4 m/s) and right to left (RL) shunt during the diastolic period (rate, 2.4 m/s). The ratio of pulmonary artery acceleration time/right ventricular ejection time was 0.23, indicating systemic pulmonary hypertension. Shortly before the operation, cardiologists confirmed that no remarkable change was found in echocardiography.

His heart rate was 130 bpm and his blood pressure was 100/60 mmHg. The patient’s hemoglobin-oxygen saturation (SpO2) was 90–93 %. The preoperative hemoglobin concentration was 13.8 g/dl, with a hematocrit of 40.8 %.

The electrocardiogram showed a sinus rhythm with right axis deviation and incomplete right bundle blanch block.

**Fig. 1:**
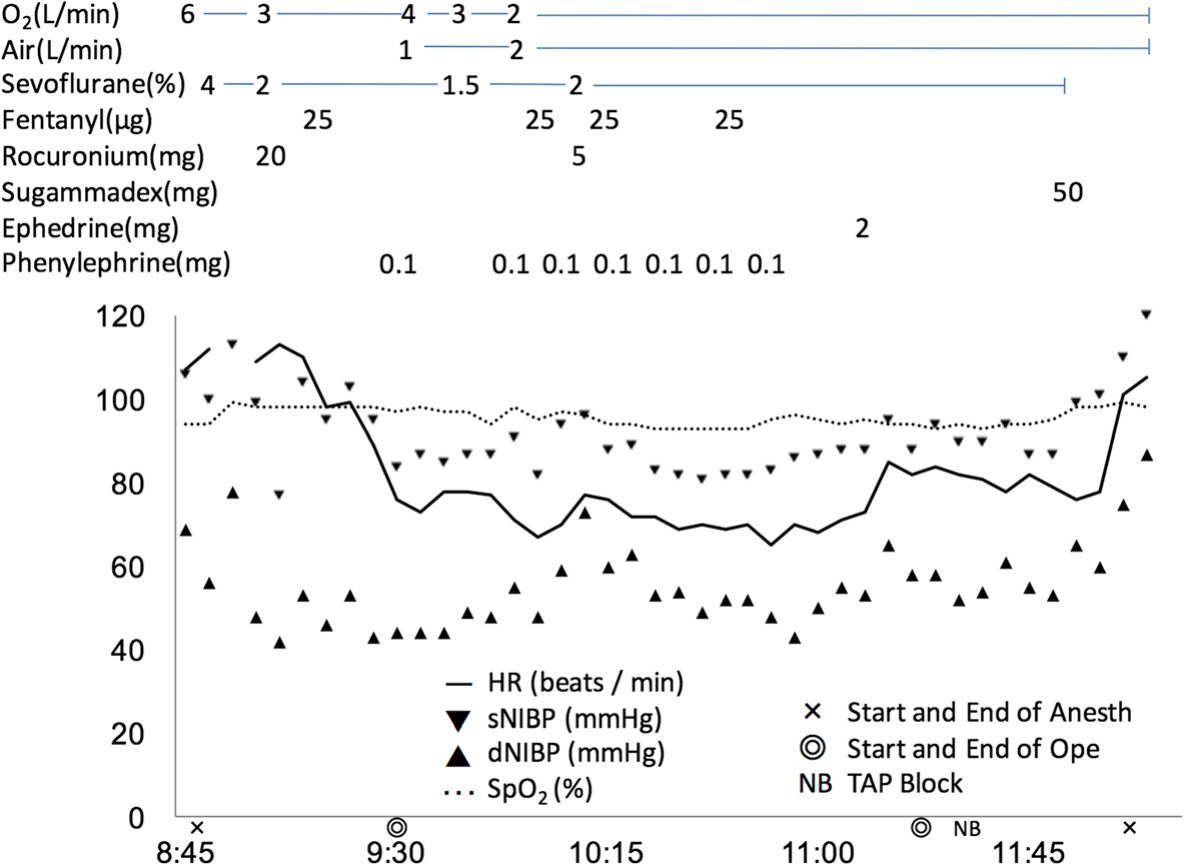
Anesthesia timeline record. Perioperative hemodynamics was stable and anesthesia was used uneventfully in this patient. Phenylephrine (0.025 mg) was administered intravenously when it was needed to maintain blood pressure

The anesthesia timeline is shown in Fig. 1. Electrocardiogram, pulse oximetry, and oscillometry were monitored when the patient entered the operating room. Anesthesia was carefully induced with 4 % sevoflurane in 100 % oxygen through the tracheostomy orifice and was maintained with 2 % sevoflurane. During anesthesia induction, a right posterior tibial arterial line was secured for invasive arterial pressure monitoring and blood gas analysis. Blood gas analysis were performed twice and showed no remarkable changes. Venous access was also established in his left arm. Intravenous fentanyl (25 μg) and rocuronium (20 mg) were administered before the start of the operation.

**Fig. 2:**
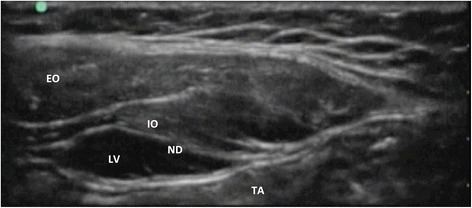
TAP block was performed bilaterally with ultrasound echo. Three layers of muscles forming the anterior abdominal wall, from superficial to deep: external oblique (EO), internal oblique (IO), and transversus abdominus (TA). A 22-G Tuohy block needle (ND) was advanced directly towards the TA. Levobupivacaine (LV) (0.25 %) was administered at a total of 10 ml on each side of TA

The surgery was uneventful. Perioperative hemodynamics was stable as shown in Fig. 1. Fentanyl (25 μg) was administered three times during the operation and phenylephrine (0.025 mg) was administered when needed to maintain blood pressure. The duration of the surgery was 124 min. The blood loss was 5 ml, with infusion of 300 ml of crystalloid, during the operation. Immediately after surgery, a bilateral TAP block was performed under ultrasound guidance (SonoSite M-Turbo, P12645-02). A 22-G Tuohy block needle (Unilever, Unisys, Saitama, Japan) was advanced directly towards the TAP. Levobupivacaine (5 mL of 0.25 %; Maruishi Pharm. Co., Osaka Japan) was administered into each side (Fig. 2). Sevoflurane inhalation was discontinued when the block was completed. The patient recovered from anesthesia without circulatory or respiratory problems. The use of the patient’s artificial ventilator was resumed with the former setting, and he was transferred to the recovery room. The postoperative pain relief was satisfactory overnight, without any requirement for additional analgesic agent. He was discharged from the hospital 3 days later.

Eisenmenger’s syndrome is characterized by the process in which a LR shunt due to a congenital heart disease, such as atrial or ventricular septal defect or patent ductus arteriosus, causes increased pulmonary blood flow; this leads to increased pulmonary vascular resistance to a level that equals or exceeds the systemic vascular resistance, causing reversal of the LR shunt to RL shunt. A promising treatment has not been indicated for decreasing pulmonary vascular resistance in patients with this type of cyanotic heart disease because the pulmonary vascular resistance is nearly fixed and the response to vasodilators or oxygen is minimal. Surgical repair of the RL shunt is not indicated because it would worsen right ventricular failure, which often results in death. Perioperative management of patients with Eisenmenger’s syndrome is one of the greatest concerns for the anesthesiologist [1–4].

Because patients with Eisenmenger’s syndrome are at high risk of perioperative mortality, non-cardiac surgery should only be performed in cases in which it is deemed absolutely essential [3, 6–9].

The following factors are considered predictors of poor prognosis: syncope, age of onset or development of syndrome, elevated right atrial pressure, supraventricular arrhythmia, poor New York Heart Association (NYHA) class, severe right ventricular dysfunction, renal failure, severe hyperemia (SpO2 < 85 %), and trisomy 21 [4, 6, 10].

To our knowledge, evidence-based guidelines do not exist for anesthetic pain management for patients with Eisenmenger’s syndrome during the perioperative period. Thus, anesthesiologists should be familiar with the specification of the patient’s anatomical and physiological conditions. In any case, the fundamental principle of anesthetic management in patients with Eisenmenger’s syndrome is to minimize increases in pulmonary vascular resistance (PVR) and to maintain systemic vascular resistance (SVR). Increased PVR and decreased SVR lead to exacerbation of RL shunting, resulting in hypoxemia, severe bradycardia, and, occasionally, cardiac arrest. Although some feasible methods are available for preventing PH crisis, such as hyperventilation with 100 % O2 and inhalation of nitric oxide, PVR in patients with Eisenmenger’s syndrome may be unresponsive to these therapies [11].

In this case, general anesthesia was utilized because the patient was too young to undergo the surgery only with regional anesthesia. During the operation, the systolic arterial pressure was maintained over 70 mmHg by using phenylephrine and paying attention to intravascular volume. We successfully managed the circulatory conditions and kept the SpO2 over 90 % while using anesthesia.

Since hypercapnia, excitement, and emotional stress are also critical factors associated with deterioration of hemodynamics and death during the perioperative period in patients with Eisenmenger’s syndrome, the use of peripheral nerve block for postoperative pain relief is indicated [5, 12, 13].

TAP block is a highly beneficial method for postoperative pain management. The incidence of overall complications with TAP block in children was equal to or less than 0.3 % in children. Circulatory depression was considered lower in TAP block than in neuroaxial regional techniques of epidural and spinal anesthesia [14]. Although the application of TAP block to a patient with Eisenmenger’s syndrome has not been reported, TAP block was performed safely in our case by using ultrasound devices.

Inguinal hernia repair is commonly performed under general anesthesia combined with an ilioinguinal iliohypogastric nerve (IHN) block. Data are available in which IHN block or local anesthetic infiltration was compared with ultrasound-guided TAP block in patients undergoing inguinal hernia repair. The reports indicate that ultrasound-guided TAP block provided the same or better pain control than IHN block or local anesthetic infiltration after inguinal hernia repair [15, 16]. The positions of the skin incision are nearly identical for inguinal hernia repair and orchiopexy. Thus, we selected TAP block for postoperative analgesia, and we completed this technique safely in the patient.

## Conclusion

We have presented a case in which a TAP block was performed for postoperative analgesia in an 8-year-old child with Eisenmenger’s syndrome undergoing bilateral orchiopexy surgery. He had good postoperative pain relief, with maintenance of hemodynamics during the perioperative period.

## Consent

Written informed consent was obtained from the patient’s parent for publication of this Case report and any accompanying images. A copy of the written consent is available for review by the Editor-in-Chief of this journal.

## Abbreviations

IHN: Ilioinguinal iliohypogastric nerve
LR: Left to right
mVSD: Muscular ventricular septal defect
NYHA: New York Heart Association
PVR: Pulmonary vascular resistance
RL: Right to left
SVR: Systemic vascular resistance
TAP: Transversus abdominis plane
